# Adaptive Compressive Sensing of Images Using Spatial Entropy

**DOI:** 10.1155/2017/9059204

**Published:** 2017-10-22

**Authors:** Ran Li, Xiaomeng Duan, Xiaoli Guo, Wei He, Yongfeng Lv

**Affiliations:** ^1^School of Computer and Information Technology, Xinyang Normal University, Xinyang 464000, China; ^2^School of Media, Xinyang Normal University, Xinyang 464000, China

## Abstract

Compressive Sensing (CS) realizes a low-complex image encoding architecture, which is suitable for resource-constrained wireless sensor networks. However, due to the nonstationary statistics of images, images reconstructed by the CS-based codec have many blocking artifacts and blurs. To overcome these negative effects, we propose an Adaptive Block Compressive Sensing (ABCS) system based on spatial entropy. Spatial entropy measures the amount of information, which is used to allocate measuring resources to various regions. The scheme takes spatial entropy into consideration because rich information means more edges and textures. To reduce the computational complexity of decoding, a linear mode is used to reconstruct each block by the matrix-vector product. Experimental results show that our ABCS coding system provides a better reconstruction quality from both subjective and objective points of view, and it also has a low decoding complexity.

## 1. Introduction

Compressive Sensing (CS) is a novel sampling theory that goes against the conventional Nyquist-Shannon theorem in data acquisition [1]. When married with image coding, CS brings a low-complex encoding architecture, which is appealing for resource-constrained wireless sensor network [2]. Image CS coding is to reconstruct the natural image from its observed measurements **y** = Φ**x**, where **x** ∈ **R**^*N*^ is lexicographically stacked representations of the original image and **y** ∈ **R**^*M*^ is the CS measurements observed by a random *M* × *N* measurement matrix Φ(*M* ≪ *N*). Once the image **x** is* K*-sparse signal (*K* ≪ *N*) in some space Ψ, CS theory can guarantee that the image is accurately recovered with high probability from *M* = *O*(*K*log⁡*N*) measurements [3]. The CS measurement process combines image acquisition and image compression; thus the computational burdens are greatly reduced at encoder. Each element in **y** carries equal amount of the information on **x**, which offers a robust ability against noise in wireless communication. The advantages of CS attract many researchers to explore applications of CS in multimedia system [4, 5].

Many researchers have been attempting to develop effective image reconstruction algorithms in order to improve the rate-distortion performance of image CS coding. A good reconstruction performance relies on a more sparse representation of image; for example, Zhang et al. [8] exploit the intrinsic local sparsity and nonlocal self-similarity to design a dynamically varying space; Wu et al. [9] introduce a local autoregressive model to explore sparse components; Eslahi et al. [10] construct an adaptively learned space by using local and nonlocal sparsity of image; Liu et al. [11] use Principle Component Analysis (PCA) to sparsely decompose each patch in image. In the field of Magnetic Resonance Imaging (MRI), some works also invest many efforts to improve the reconstruction performance; for example, Zhang et al. [12] proposed an energy preserving sampling to enhance the quality of digital phantom, Zhang et al. [13] proposed an exponential wavelet iterative shrinkage/threshold algorithm to reduce the blurs existing in the reconstructed image, and Sun and Gu [14] proposed an adaptive observation matrix for sparse samples for ultrasonic wave signals that are analyzed in the phased array structural health monitoring. The above-mentioned methods all involve numerical iteration, which brings a high computational complexity at decoder. Therefore, the image CS coding is always characterized by light encoding and heavy decoding. However, because natural images typically exhibit nonstationary statistics, high computational complexity does not necessarily bring a satisfactory result. That poses us a challenge about how to design a CS codec system which can overcome the negative effects of nonstationary statistics.

Block-based CS (BCS) hybrid coding framework [15–17] solves the problem of high computational complexity of decoding by measuring and recovering nonoverlapping blocks independently, but nonstationary statistics of image could lead to blocking artifacts. Different statistics of block result in different sparsity of block; thus the measurement times of block should be set accordingly. Based on BCS framework, some research on Adaptive BCS (ABCS) framework [6, 7, 18] is done to suppress blocking artifacts. The research all uses some image features (e.g., DCT coefficient [18], variance [6], and saliency [7]) to measure statistics of block and then adaptively allocates CS measurements for each block according to the measured feature of block. ABCS is a successful scheme to reduce the negative effect of nonstationary statistics while guaranteeing a low computational complexity of decoding. However, some time and space complexities would inevitably be introduced at encoder due to the existence of feature exaction. The existing ABCS schemes invest many matrix-vector products to compute image feature; for example, two matrix-vector products and one convolution operation are performed for the whole image to compute the visual saliency in [7]. The matrix-vector product is too expensive for wireless sensor network because the processor of mobile note has limited computing capability. Therefore, in order to make encoder lighter, ABCS framework requires a simple feature while effectively reducing blocking artifacts.

In this paper, we propose an ABCS coding system which uses spatial entropy of block to allocate measuring resources. Spatial entropy measures the amount of information, revealing statistical characteristic of data. The main contributions of this work can be summarized as follows:

- We propose using the spatial entropy of image block as a criterion of CS measurements allocation.
- We reduce the computational complexity of reconstructing image by using a linear model.

We assign higher measurement rate to blocks with much information but lower measurement rate to blocks with less information. By entropy-based adaptive measuring, the quality of reconstructed block could not vary greatly with nonstationary statistics of image. Since the computing of entropy requires only a few floating-point operations, our ABCS system also has a light encoder. To realize real-time decoding, we use a linear model to recover all blocks. Combined with adaptive measuring based on spatial entropy, the linear recovery method improves the reconstruction quality effectively.

The rest of this paper is organized as follows.  Section 2 summarizes ABCS coding framework.  Section 3 presents the proposed adaptive measuring and linear recovery schemes. Experimental results are given in Section 4 and conclusion in Section 5.

## 2. ABCS Coding Framework

The advantage of ABCS framework is nonuniform allocation of CS measurements based on the image feature. This section shows how ABCS framework works.

**Figure 1:**
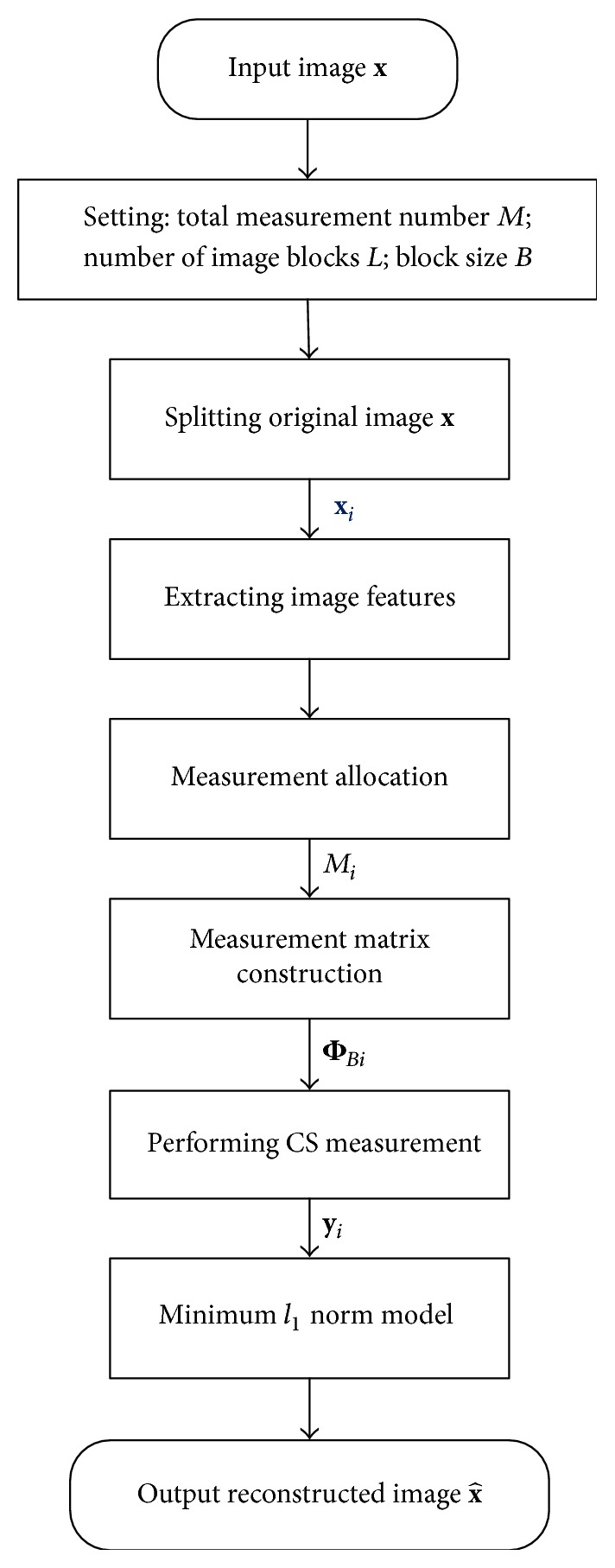
Flow of ABCS coding.

Given an *N*-pixel image **x** from a real-world scene and supposing we want to take *M* CS measurements, we summarize the flow of ABCS coding, as shown in Figure 1. The encoding part is described as follows.

**Step 1 .**

Divide image **x** into *L* nonoverlapping blocks of *B* × *B* in size and let **x**_*i*_ (*i* = 1,2,…, *L*) represent the vectorized signal of the *i*th block through raster scanning.

**Step 2 .**

The feature of each block is extracted. Block variance [18], edge [6], and saliency information [7] are common features.

**Step 3 .**

We set the measurement number *M*_*i*_ of each block according to the distribution of these image features. The total number of CS measurements of all blocks is *M*; that is, ∑_i=1_^L^M_i_ = *M*.

**Step 4 .**

We use Marsaglia's ziggurat algorithm [19] to produce pseudorandom data which obey Gaussian distribution, and these random data form a *B*^2^ × *B*^2^ matrix Θ. After that, we randomly pick *M*_*i*_ rows from Θ to construct the *M*_*i*_ × *B*^2^ measurement matrix Φ_*Bi*_ of **x**_*i*_.

**Step 5 .**

The CS measurement vector **y**_*i*_ of **x**_*i*_ is observed with Φ_*Bi*_ as follows:

(1) $$\begin{array}{c} y_{i}=\Phi_{Bi}x_{i}. \end{array}$$

We define the block measurement rate *R*_*i*_ as *M*_*i*_/*B*^2^.

Through the above steps, we perform ABCS encoding for an image. According to (1), the measurement rate of each block varies with different image features. By measuring block features, more CS measurements are allocated to blocks with high-level features but fewer to blocks with low-level features.

At the ABCS decoder, after receiving the measurement vector **y**_*i*_ of each block, ABCS framework generally uses the minimum *l*_1_ norm model to recover each block as follows:

(2) x^i=arg⁡min Ψixi1s.t. yi−ΦBixi2≤ε

in which ‖·‖_1_ and ‖·‖_2_ are *l*_1_ and *l*_2_ norms, respectively, Ψ_*i*_ is the transformation matrix of each block, for example, DCT and wavelet matrices, and *ε* is the noise tolerance which can be set based on experience. Model (2) can be solved by many numerical iterative algorithms, for example, Orthogonal Matching Pursuit (OMP) [20] and Gradient Projection for Sparse Reconstruction (GPSR) [21]. These algorithms require a high computational complexity to reconstruct a whole image. No matter what recovery algorithm is chosen, more CS measurements mean a better reconstruction quality. Therefore, ABCS framework ensures a good recovery quality for every block by feature-based adaptive measuring.

## 3. Proposed Scheme

**Figure 2:**
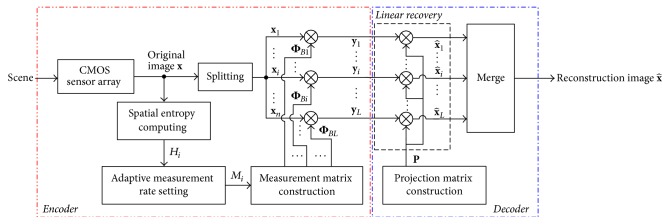
Proposed ABCS framework.

Figure 2 presents the framework of the proposed ABCS scheme. At encoder, we compute the spatial entropy *H*_*i*_ of the *i*th block **x**_*i*_ and set its measurement number *M*_*i*_ according to the distribution of spatial entropy. We construct the *M*_*i*_ × *B*^2^ measurement matrix Φ_*Bi*_ to observe CS measurement vector **y**_*i*_. Spatial entropy measures the information amount of each block and directly reveals the nonstationary statistics of image. By entropy-based adaptive measuring, each block has sufficient CS measurements to describe the block statistics. At decoder, in order to realize real-time decoding, we transform the measurement vector **y**_*i*_ into the reconstructed block ${\hat{\text{x}}}_{\text{i}}$ by a linear model. In the following three parts of this section, we first describe how to compute the distribution of spatial entropy, then design an adaptive measuring scheme, and finally present the linear recovery model.

### 3.1. Spatial Entropy

Spatial entropy of image is the expected value of the information contained in some pixels. We compute the spatial entropy *H*_*i*_ of the *i*th block as follows:

(3) $$\begin{array}{c} H_{i}=-\overset{255}{\underset{j=0}{\sum }}p_{ij}\;\log_{2}p_{ij}, \end{array}$$

in which *j* represents pixel value and *p*_*ij*_ is the probability of pixel value in **x**_*i*_. The unit of *H*_*i*_ is bit per pixel (bpp), and *H*_*i*_ is the minimum number of bits to encode any pixel in a block with no loss. Data processing inequality states that the information content of a signal cannot be increased via a local physical operation [20], which implies that the information contained in sparse components is close to spatial entropy. Therefore, the bigger the spatial entropy of block is, the less sparse the representation coefficients are, and vice versa. According to CS theory, we should allocate more CS measurements to blocks with much information but fewer to blocks with less information. By normalizing the spatial entropy of each block,

(4) $$\begin{array}{c} w_{i}=\frac{H_{i}}{\sum_{i=1}^{L}H_{i}}, \end{array}$$

we can control the measurement rate *R*_*i*_ according to the entropy contrast. The probabilities can be expressed in the form of histograms; thus the spatial entropies of all blocks can be computed in *O*(*N*) time order.

### 3.2. Measuring Allocation

Our entropy-based CS scheme aims to allocate measuring resources according to the information contained in each block. By (4), we obtain the distribution of spatial entropy. Suppose *M* is the total number of CS measurements for a whole image, we set the number of CS measurements for each block as follows:

(5) $$\begin{array}{c} M_{i}=round\left[\;w_{i}\left(\;M-LM_{0}\;\right)+M_{0}\;\right], \end{array}$$

in which *M*_0_ is the initial measurement number of each block and round[·] is the round operation. By (1), the excessive CS measurements are assigned to blocks with much information. BCS allocates measuring resources equally to all blocks because it cannot tell how much information it contains and differentiate one from another. Our scheme takes into account the statistics of image. By exploiting the spatial entropy of each block, the scheme allocates more random measurements to rich-information blocks but fewer to poor-information blocks. The CS theory states that a recovery algorithm would offer better reconstruction quality of a block with more measurements. Therefore, when using the same number of measurements for the whole image, our entropy-based scheme can better recover blocks with much information compared to BCS.

### 3.3. Linear Recovery

Conventional CS recovery algorithms use numerical calculation to nonlinearly reconstruct the image. The numerical calculation involves loop iteration, introducing a high computational complexity. Therefore, the conventional recovery algorithm is not suitable for the real-time decoding. Equation (1) indicates that the measurement vector **y**_*i*_ is a projection of **x**_*i*_ onto a low-dimensional space; thus there is a linear relation between **y**_*i*_ and **x**_*i*_. By using the linear relation, we can design a projection matrix **P** to back-project **y**_*i*_ onto the neighboring region of **x**_*i*_; that is,

(6) $$\begin{array}{c} {\hat{x}}_{i}=Py_{i} \end{array}$$

in which ${\hat{\text{x}}}_{\text{i}}$ is the linear estimation of **x**_*i*_. From the above, the linear recovery consists of two steps: learning a projection matrix **P** and reconstructing each block by using the matrix **P**. We first describe how to learn the projection matrix **P**. The error vector **e** between ${\hat{\text{x}}}_{\text{i}}$ and **x**_*i*_ can be computed as follows:

(7) $$\begin{array}{c} e_{i}=x_{i}-{\hat{x}}_{i}=x_{i}-Py_{i}. \end{array}$$

We should select a projection matrix **P** to minimize the error vector **e**_*i*_. Based on this motivation, we design an optimization model to choose the best projection matrix as follows:

(8) Popt=arg⁡minP·Ree=EeieiT=Exi−Pyixi−PyiT,

in which **R**_*ee*_ is autocorrelation function of **e**_*i*_ and *E*(·) is the expectation function. Setting the gradient of **R**_*ee*_ (with respect to **P**) to** 0**, we can obtain the solution of model (8) as

(9) $$\begin{array}{c} P_{opt}=R_{xy}R_{yy}^{-1}=E\left[\;x_{i}y_{i}^{T}\;\right]E^{-1}\left[\;y_{i}y_{i}^{T}\;\right]. \end{array}$$

Plug (1) into (9) and we get

(10) $$\begin{array}{c} P_{opt}=E\left[\;x_{i}{\left(\;\Phi_{Bi}^{}x_{i}\;\right)}^{T}\;\right]E\left[\;\Phi_{Bi}^{}x_{i}{\left(\;\Phi_{Bi}^{}x_{i}\;\right)}^{T}\;\right]. \end{array}$$

Because Φ_*Bi*_ is a known matrix, we can move it to the outside of *E*[·]; that is,

(11) $$\begin{array}{c} P_{opt}=E\left[\;x_{i}{x_{i}}^{T}\;\right]\Phi_{Bi}^{T}\Phi_{Bi}^{}E\left[\;x_{i}{x_{i}}^{T}\;\right]\Phi_{Bi}^{T}. \end{array}$$

Let

(12) $$\begin{array}{c} R_{xx}=E\left[\;x_{i}x_{i}^{T}\;\right], \end{array}$$

in which we regard **x**_*i*_ as a random vector, and **R**_*xx*_ is autocorrelation function of **x**_*i*_. That is,

(13) $$\begin{array}{c} R_{xx}=\left[\begin{array}{cccc} E\left(x_{i1}x_{i1}\right) & E\left(x_{i1}x_{i2}\right) & \cdots & E\left(x_{i1}x_{iB^{2}}\right)\\ E\left(x_{i2}x_{i1}\right) & E\left(x_{i2}x_{i2}\right) & \cdots & E\left(x_{i2}x_{iB^{2}}\right)\\ \vdots & \vdots & \ddots & \vdots \\ E\left(x_{iB^{2}}x_{i1}\right) & E\left(x_{iB^{2}}x_{i2}\right) & \cdots & E\left(x_{iB^{2}}x_{iB^{2}}\right) \end{array}\right]. \end{array}$$

It is difficult to directly compute each element of **R**_*xx*_, but we can estimate it by the following statistic model:

(14) Rxxm,n=EximxinT=ρδm,n,δm,n=dist⁡xim,xin=m1−m2+n1−n2,

in which (*m*_1_, *n*_1_) is the spatial position of pixel *x*_*im*_ and (*m*_2_, *n*_2_) is the spatial position of pixel *x*_*in*_. *δ*_*m*,*n*_ is the chessboard distance between *x*_*im*_ and *x*_*in*_. *ρ* is a constant between 0.9 and 1, and we set *ρ* to be 0.95 by experience. Through the above operations, we obtain the best projection matrix **P**_opt_, and then each block can be recovered by

(15) $$\begin{array}{c} {\hat{x}}_{i}=P_{opt}y_{i}. \end{array}$$

The flow of linear image recovering is summed in Algorithm 1.

Through this matrix-vector product for each image block, we can get the estimation of the original block. Divide an image into *L* nonoverlapping blocks, use matrix-vector product for *L* times, and we can achieve the reconstruction of the whole image. The total computation is *M* × *B*^2^ multiplications and *M* × *B*^2^ additions, which is far less than that of conventional CS recovery algorithm.

## 4. Experimental Results

We evaluate the performance of our ABCS coding system on a number of grayscale images of 512 × 512 in size including* Lenna*,* Barbara*,* Peppers*,* Goldhill,* and* Mandrill*. These reconstructed images by our system are compared with those by conventional BCS system [15], variance-based ABCS (V-ABCS) system [6], and saliency-based ABCS (S-ABCS) system [7] from subjective and objective points of view. These compared schemes use OMP algorithm [20] to nonlinearly recover all blocks. In all experiments, the block size *B* is set to be 16, and we set the total measurement rate *R* (=*M*/*N*) to be between 0.1 and 0.5. PSNR in dB and Structure SIMilarity (SSIM) [22] between the reconstructed image and the original image are used in the objective evaluation. All experiments are conducted under the following computer configuration: Intel(R) Core (TM) i7 @ 3.30 GHz CPU, 8 GB RAM, Microsoft Windows 7 64 bits, and MATLAB Version 7.6.0.324 (R2008a).

### 4.1. Subjective Evaluation

**Figure 3:**
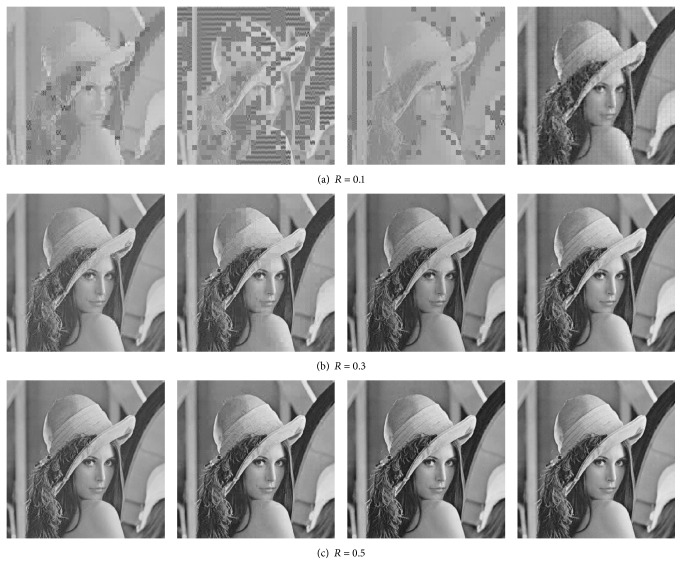
Subjective comparison of reconstructed* Lenna* images by various CS-based codec at different measurement rates. From left to right: BCS, V-ABCS, S-ABCS, and the proposed ABCS. Note that *R* is the total measurement rate.

**Figure 4:**
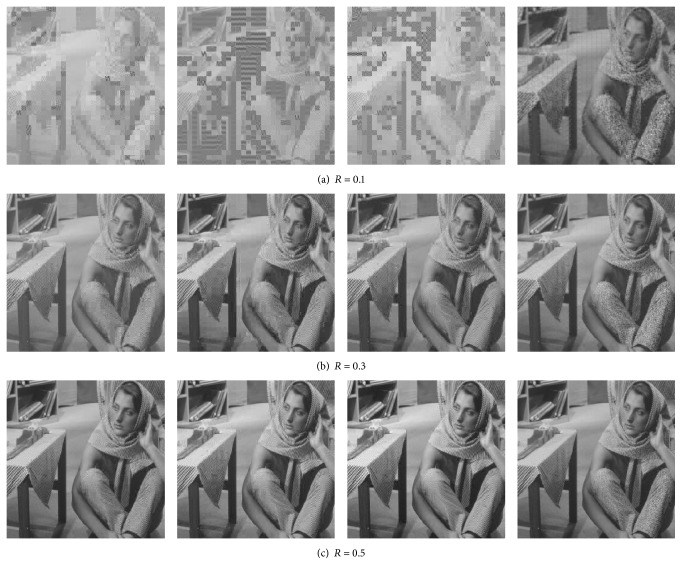
Subjective comparison of reconstructed* Barbara* images by various CS-based codec at different measurement rates. From left to right: BCS, V-ABCS, S-ABCS, and the proposed ABCS. Note that *R* is the total measurement rate.

**Figure 5:**
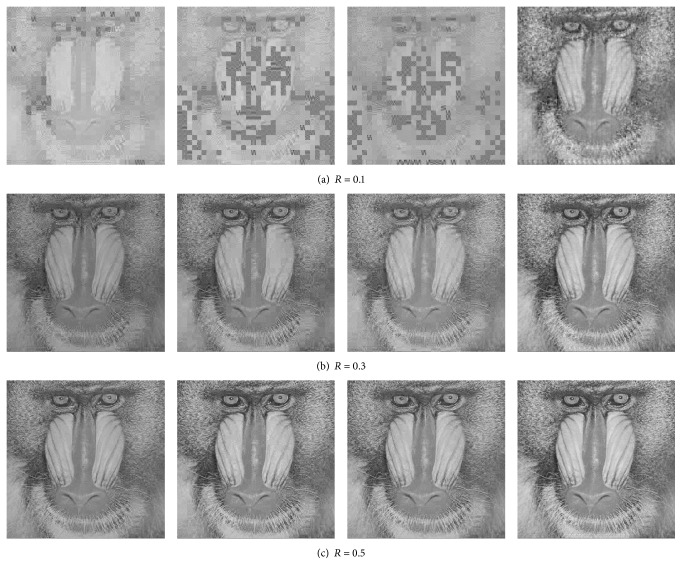
Subjective comparison of reconstructed* Mandrill* images by various CS-based codec at different measurement rates. From left to right: BCS, V-ABCS, S-ABCS, and the proposed ABCS. Note that *R* is the total measurement rate.

Figures 3, 4, and 5 present the visual reconstruction results of* Lenna*,* Barbara,* and* Mandrill* by various CS-based codecs at different measurement rates. When measurement rate *R* is 0.1, the CS measurements of each block are not enough to guarantee the convergence of OMP algorithm for BCS, V-ABCS, and S-ABCS systems; thus lots of reconstructed blocks lose structural details. The reconstructed images by our ABCS system have better surfaces and edges of objects, but there are many blocking artifacts in a whole image. As the measurement rate increases, the reconstructed images by BCS, V-ABCS, and S-ABCS systems are improved significantly, but there are still many blocking artifacts, and some blurs occur in the region of edges and textures. Although it cannot better recover texture details (e.g., periodic stripes near trouser legs in* Barbara*), our system effectively reduces blurs in edge regions. For* Mandrill* with lots of hairs, our system also recovers finer hairs than those of other systems at any measurement rate. On the whole, we can see that our ABCS system can guarantee a better visual quality.

### 4.2. Objective Evaluation

Table 1 compares PSNR for test images at the measurement rate of 0.1, 0.3, and 0.5, respectively. The results indicate that our ABCS system achieves the highest average PSNR values for all test images at any measurement rate; for example, when the measurement rate *R* is 0.1, our system is 5.18 dB on average higher than S-ABCS for* Lenna*. For* Barbara*, our system cannot obtain higher PSNR than other systems at the measurement rate of 0.3 and 0.5, resulting from its limited ability to recover periodic patterns.  Table 2 presents SSIM values for test images at the measurement rate of 0.1, 0.3, and 0.5. We can see that our system outperforms other systems in most cases. For* Lenna*, our system is 0.2649, 0.0785, and 0.0396 on average higher than S-ABCS at the measurement rate of 0.1, 0.3, and 0.5, respectively. There is still SSIM degradation for our system when reconstructing* Barbara* at a high measurement rate.  Table 3 lists the average reconstruction time of various systems for all test images at the measurement rate of 0.1 to 0.5. We can see that our system requires only 1.74 s on average to reconstruct a 512 × 512 image, while other systems need about 5 s on average. The execution time of our system increases with the rising measurement rate, but only slightly. From the above, we can see that our ABCS system provides a better objective quality while guaranteeing a low computational complexity.

## 5. Conclusion

In this paper, we propose an ABCS system that adaptively measures each block according to spatial entropy and reconstructs images using a linear model. Spatial entropy reveals the variation of block sparse degree and is a simple feature revealing statistics of image. Based on the distribution of spatial entropy, we observe image blocks at different measurement rates. The entropy-based measuring reduces the redundancy of block measurements. To reduce the computational complexity of decoding, we adopt a linear model to reconstruct each block. Experimental results show that our ABCS system improves the quality of reconstructed image from both subjective and objective points of view while guaranteeing a low computational complexity.

As the research in this paper is exploratory, there are many intriguing questions that our future work should consider. First, the theory of adaptive block CS needs to be developed. Second, the entropy computation in the measurement domain is the target in our future work. And last, we hope to extend this work to CS of color images and videos.

## Figures and Tables

**Algorithm 1:**
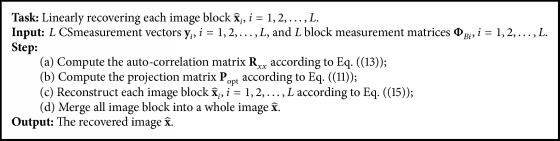
The flow of linear image recovering.

**Table 1:** PSNR (dB) comparison of various CS-based codec for test images at different measurement rates.

Test image	BCS		V-ABCS [6]		S-ABCS [7]		Proposed
	PSNR	ΔPSNR	PSNR	ΔPSNR	PSNR	ΔPSNR	PSNR
*R* = 0.1							
*Lenna*	18.89	−8.19	18.58	−8.50	19.72	−7.36	27.08
*Barbara*	16.64	−5.29	17.02	−4.91	17.05	−4.88	21.93
*Peppers*	17.28	−9.10	17.46	−8.92	18.31	−8.07	26.38
*Goldhill*	20.49	−5.59	20.47	−5.61	21.24	−4.84	26.08
*Mandrill*	15.71	−3.93	18.14	−1.50	18.89	−0.75	19.64
Avg.	17.80	−6.42	18.33	−5.89	19.04	−5.18	24.22

*R* = 0.3							
*Lenna*	27.35	−5.34	29.05	−3.64	30.54	−2.15	32.69
*Barbara*	24.02	−0.81	25.60	0.77	25.94	1.11	24.83
*Peppers*	24.35	−6.96	28.59	−2.72	29.34	−1.97	31.31
*Goldhill*	23.86	−6.49	26.70	−3.65	27.08	−3.27	30.35
*Mandrill*	17.64	−5.13	19.74	−3.03	19.39	−3.38	22.77
Avg.	23.44	−4.95	25.94	−2.45	26.46	−1.93	28.39

*R* = 0.5							
*Lenna*	31.64	−4.6	32.10	−4.14	34.41	−1.83	36.24
*Barbara*	28.35	0.70	29.27	1.62	30.69	3.04	27.65
*Peppers*	31.11	−3.01	31.18	−2.94	32.70	−1.42	34.12
*Goldhill*	29.19	−4.1	29.19	−4.1	30.61	−2.68	33.29
*Mandrill*	21.04	−4.31	22.76	−2.59	22.82	−2.53	25.35
Avg.	28.27	−3.06	28.90	−2.43	30.25	−1.08	31.33

**Table 2:** SSIM comparison of various CS-based codec for test images at different measurement rates.

Test image	BCS		V-ABCS [6]		S-ABCS [7]		Proposed
	SSIM	ΔSSIM	SSIM	ΔSSIM	SSIM	ΔSSIM	SSIM
*R* = 0.1							
*Lenna*	0.5903	−0.2387	0.5447	−0.2843	0.5169	−0.3121	0.8290
*Barbara*	0.4823	−0.2299	0.5886	−0.1236	0.5618	−0.1504	0.7122
*Peppers*	0.5589	−0.2700	0.5521	−0.2768	0.5141	−0.3148	0.8289
*Goldhill*	0.5314	−0.2392	0.5218	−0.2488	0.5037	−0.2669	0.7706
*Mandrill*	0.3312	−0.259	0.3094	−0.2808	0.3100	−0.2802	0.5902
Avg.	0.4988	−0.2474	0.5033	−0.2429	0.4813	−0.2649	0.7462

*R* = 0.3							
*Lena*	0.8850	−0.0696	0.8556	−0.099	0.9082	−0.0464	0.9546
*Barbara*	0.8482	−0.0171	0.8292	−0.0361	0.8630	−0.0023	0.8653
*Peppers*	0.8826	−0.0607	0.8462	−0.0971	0.8939	−0.0494	0.9433
*Goldhill*	0.8207	−0.1055	0.7939	−0.1323	0.8334	−0.0928	0.9262
*Mandrill*	0.6334	−0.1995	0.6285	−0.2044	0.6312	−0.2017	0.8329
Avg.	0.8140	−0.0905	0.7907	−0.1138	0.8259	−0.0785	0.9045

*R* = 0.5							
*Lenna*	0.9515	−0.0285	0.9170	−0.063	0.9570	−0.023	0.9800
*Barbara*	0.9367	0.0044	0.9125	−0.0198	0.9433	0.011	0.9323
*Peppers*	0.9412	−0.0273	0.9029	−0.0656	0.9422	−0.0263	0.9685
*Goldhill*	0.9164	−0.0512	0.8722	−0.0954	0.9218	−0.0458	0.9676
*Mandrill*	0.7964	−0.1239	0.7843	−0.136	0.8066	−0.1137	0.9203
Avg.	0.9084	−0.0453	0.8778	−0.0760	0.9142	−0.0396	0.9537

**Table 3:** Average reconstruction time (s) of various CS-based codec for all test images at different measurement rates.

Measurement rate	BCS	V-ABCS [6]	S-ABCS [7]	Proposed
0.1	2.81	3.14	3.08	0.91
0.2	3.51	4.09	4.05	1.23
0.3	4.31	5.10	5.05	1.67
0.4	5.13	6.14	6.18	2.16
0.5	6.02	7.08	7.22	2.74
Avg.	4.36	5.11	5.12	1.74

## References

[1] Donoho D. L. (2006). Compressed sensing. *Institute of Electrical and Electronics Engineers Transactions on Information Theory*.

[2] Wakin M. B., Candes E. J. (2008). An introduction to compressive sensing. *IEEE Signal Processing Magazine*.

[3] Candes E. J., Romberg J., Tao T. (2006). Robust uncertainty principles: exact signal reconstruction from highly incomplete frequency information. *Institute of Electrical and Electronics Engineers Transactions on Information Theory*.

[4] Yuan X., Wang X., Wang C., Weng J., Ren K. (2016). Enabling secure and fast indexing for privacy-assured healthcare monitoring via compressive sensing. *IEEE Transactions on Multimedia*.

[5] Song X., Peng X., Xu J., Shi G., Wu F. (2017). Distributed compressive sensing for cloud-based wireless image transmission. *IEEE Transactions on Multimedia*.

[6] Zhang J., Xiang Q., Yin Y., Chen C., Luo X. (2017). Adaptive compressed sensing for wireless image sensor networks. *Multimedia Tools and Applications*.

[7] Yu Y., Wang B., Zhang L. (2010). Saliency-based compressive sampling for image signals. *IEEE Signal Processing Letters*.

[8] Zhang J., Zhao D., Gao W. (2014). Group-based sparse representation for image restoration. *IEEE Transactions on Image Processing*.

[9] Wu X., Dong W., Zhang X., Shi G. (2012). Model-assisted adaptive recovery of compressed sensing with imaging applications. *IEEE Transactions on Image Processing*.

[10] Eslahi N., Aghagolzadeh A., Andargoli S. M. H. (2016). Image/video compressive sensing recovery using joint adaptive sparsity measure. *Neurocomputing*.

[11] Liu X., Zhai D., Zhou J., Zhang X., Zhao D., Gao W. (2016). Compressive sampling-based image coding for resource-deficient visual communication. *IEEE Transactions on Image Processing*.

[12] Zhang Y., Peterson B. S., Ji G. (2014). Energy preserved sampling for compressed sensing MRI. *Computational & Mathematical Methods in Medicine*.

[13] Zhang Y., Dong Z., Phillips P., Wang S., Ji G., Yang J. (2015). Exponential wavelet iterative shrinkage thresholding algorithm for compressed sensing magnetic resonance imaging. *Information Sciences*.

[14] Sun Y., Gu F. (2017). Compressive sensing of piezoelectric sensor response signal for phased array structural health monitoring. *International Journal of Sensor Networks*.

[15] Gan L. Block compressed sensing of natural images.

[16] Mun S., Fowler J. E. Block compressed sensing of images using directional transforms.

[17] Mun S., Fowler J. E. DPCM for quantized block-based compressed sensing of images.

[18] Stankovi V., Stankovi L., Cheng S. Compressive image sampling with side information.

[19] Marsaglia G., Tsang W. W. (2000). The ziggurat method for generating random variables. * Journal of Statistical Software *.

[20] Shen Y., Li S. (2015). Sparse signals recovery from noisy measurements by orthogonal matching pursuit. *Inverse Problems and Imaging*.

[21] Figueiredo M. A. T., Nowak R. D., Wright S. J. (2007). Gradient projection for sparse reconstruction: Application to compressed sensing and other inverse problems. *IEEE Journal of Selected Topics in Signal Processing*.

[22] Wang Z., Bovik A. C., Sheikh H. R., Simoncelli E. P. (2004). Image quality assessment: from error visibility to structural similarity. *IEEE Transactions on Image Processing*.

